# Cyanidin-3-O-Glucoside: Biosynthetic Regulation, In Vivo Metabolism, and Anti-Tumor Mechanisms—An Integrated Study Based on *Sambucus nigra* L.

**DOI:** 10.3390/plants15121809

**Published:** 2026-06-12

**Authors:** Junxiu Yao, Zhengkun Cui, Xinghao Chen, Qian Zhang, Fei Ren, Xiaoman Xie

**Affiliations:** 1 Shandong Academy of Forestry, Shandong Key Laboratory of Ecological Forest & Grass Genetic Breeding, Jinan 250014, China; yjx95289528@163.com (J.Y.); zkcui@njfu.edu.cn (Z.C.); sdslkyzq@163.com (Q.Z.); lkrenfei@163.com (F.R.); 2 Shandong Key Laboratory of Eco-Environmental Science for the Yellow River Delta, Shandong University of Aeronautics, Binzhou 256603, China; chenxh60@126.com

**Keywords:** *Sambucus nigra* L., cyanidin-3-O-glucoside, antitumor, biosynthesis, transcriptional regulation, in vivo metabolism

## Abstract

*Sambucus nigra* L. (European elderberry) is distinguished among medicinal and edible plants by its exceptionally high cyanidin-3-O-glucoside (C3G) content, which markedly exceeds that of common berries. This unique phytochemical profile establishes C3G as the principal bioactive constituent underlying the antitumor activity of *S. nigra*. While numerous reviews on elderberry have been published, none has systematically integrated C3G biosynthesis, transcriptional regulation, in vivo metabolism, and anti-tumor mechanisms specifically in *S. nigra*—a critical research gap that this review fills for the first time. Herein, we systematically examine the chemical structure and content distribution of C3G in *S. nigra*, elucidate the phenylpropanoid–flavonoid biosynthetic pathway and the regulatory patterns of the MYB-bHLH-WD40 (MBW) transcriptional complex, and highlight the current research gap regarding the cloning and functional characterization of core MBW factors in this species. We further reveal the absorption and distribution characteristics of C3G in the human body, the gut microbiota-mediated biotransformation process, and the synergistic antitumor effects of its primary metabolite, protocatechuic acid. The molecular mechanisms through which C3G exerts antitumor activity, including the induction of tumor cell apoptosis, cell cycle arrest, inhibition of epithelial–mesenchymal transition, and modulation of key signaling pathways, such as NF-κB, PI3K/AKT/mTOR, and JNK, are systematically elaborated. This is the first review to construct a comprehensive “biosynthetic regulation–in vivo metabolism–antitumor function” framework for C3G in *S. nigra*, thereby addressing critical research gaps and providing a theoretical foundation for the germplasm breeding of high-C3G cultivars, functional product development, and clinical adjuvant cancer therapy.

## 1. Introduction

Malignant neoplasms constitute a major global public health challenge, characterized by steadily increasing incidence and mortality rates. The escalating limitations of conventional chemotherapeutic agents—including the development of drug resistance, severe adverse effects, and narrow therapeutic indices—have increasingly directed research efforts toward naturally derived anticancer compounds [1]. Plant-derived bioactive constituents offer unique advantages in cancer chemoprevention and adjuvant therapy, owing to their multi-targeting capabilities, low toxicity profiles, and synergistic potential [2,3]. Plant-derived polysaccharides and polyphenols have been widely demonstrated to protect against carcinogenesis by attenuating chronic oxidative damage and bolstering endogenous antioxidant defenses [4]. Among these bioactive constituents, polyphenols have garnered particular attention because their anticancer potential is intimately linked to their ability to modulate oxidative stress, which constitutes a central hallmark of cancer pathogenesis [5]. Specifically, anthocyanin polyphenols, including cyanidin-3-O-glucoside (C3G), counteract chronic oxidative damage that drives malignant transformation by scavenging reactive oxygen species (ROS), inhibiting lipid peroxidation, and enhancing endogenous antioxidant enzyme activity, thereby restoring redox homeostasis, suppressing sustained activation of pro-inflammatory and pro-survival signaling pathways, and ultimately inducing programmed cell death in transformed cells [6,7]. Against this backdrop, the anthocyanin-rich medicinal plant *Sambucus nigra* L. has garnered considerable attention in oncological research in recent years due to its remarkable pharmacological activity.

The berries of *S. nigra* are particularly rich in anthocyanins, especially C3G, with concentrations ranging from 1.12 to 5.21 mg/g FW, significantly exceeding those found in blueberries and blackberries. This distinctive feature establishes *S. nigra* as an exceptional natural source of C3G [8]. Advances in phytochemical research have led to multiple in vitro and in vivo studies confirming the marked antitumor activity of C3G, manifested through the induction of tumor cell apoptosis, cell cycle arrest, inhibition of tumor invasion and metastasis, and modulation of multiple critical signaling pathways [9,10,11,12]. Concurrently, the biosynthetic pathway of C3G in plants and its regulation by the MYB-bHLH-WD40 (MBW) transcriptional complex have been gradually elucidated [8,13], while its pharmacokinetic characteristics in humans, gut microbiota-mediated biotransformation, and bioavailability have become active areas of investigation [14,15,16]. Nevertheless, the intrinsic interconnections among these three domains remain poorly integrated. In particular, the systematic optimization of C3G content in *S. nigra* and how enhanced biosynthetic regulation might improve metabolic efficiency and ultimately potentiate antitumor efficacy remain to be rigorously investigated.

Despite extensive individual reports on the antitumor activity, synthetic regulation, and metabolic features of C3G, a comprehensive review integrating these interconnected fields is currently lacking. To address this gap, the present review aims to systematically summarize the biosynthetic pathway, transcriptional regulatory network, in vivo metabolic characteristics, and antitumor molecular mechanisms of C3G in *S. nigra*. We construct a coherent framework connecting “*S. nigra*—C3G synthetic regulation—in vivo metabolism—antitumor function” and discuss the application prospects for functional food development and adjuvant cancer therapy, thereby providing valuable insights to guide future research.

## 2. Structural Characteristics and Content Distribution of C3G in *S. nigra*

### 2.1. Chemical Structure and Physicochemical Properties of C3G

C3G belongs to the anthocyanin class of flavonoid compounds, with the molecular formula C_21_H_21_O_11_ and a molecular weight of 449.39 Da. Its core structure comprises the 2-phenylbenzopyrylium (flavylium) cation, with a glucose molecule attached via a glycosidic bond at the 3-position. This structural configuration confers potent antioxidant activity: the ortho-dihydroxyl moiety (3′,4′-dihydroxyl) on the B-ring effectively scavenges free radicals, while glucosylation enhances water solubility and stability [17]. The physicochemical properties of C3G are markedly influenced by pH. Under strongly acidic conditions (pH < 3), C3G exists as a stable red flavylium cation; as pH increases, it undergoes successive structural transformations into the colorless carbinol pseudobase, purple quinonoidal base, and blue ionized quinonoidal base [18]. These pH-dependent structural transitions not only affect color stability during food processing but also critically influence C3G stability and bioavailability across different gastrointestinal segments (acidic stomach versus neutral-to-weakly alkaline intestine), thereby presenting significant technical challenges for preserving C3G integrity from the plant source to its ultimate systemic effects.

### 2.2. C3G Content and Distribution in S. nigra

Mature berries of *S. nigra* serve as the primary natural reservoir for C3G. High-performance liquid chromatography (HPLC) quantitative analyses have demonstrated that total anthocyanin content in S. nigra berries ranges from 6.03 to 12.65 mg/g FW, with C3G accounting for 20–40% of total anthocyanins, thereby establishing it as the principal bioactive component [19]. However, advanced techniques such as UPLC-Q-TOF-MS have been shown to provide more accurate and comprehensive phytochemical profiling of bioactive compounds in medicinal plants, offering a promising complement to conventional HPLC-based methods [20]. C3G accumulation in *S. nigra* berries exhibits pronounced developmental stage dependency: anthocyanin levels are negligible in immature fruit but increase rapidly during ripening, reaching peak concentrations at full maturity [21]. Notably, substantial genetic variation in C3G content exists among different *S. nigra* genotypes, providing a robust foundation for screening and breeding elite germplasm with elevated C3G yields [22]. Additionally, *S. nigra* processing by-products retain substantial quantities of underutilized C3G. Studies have shown that pomace extracts contain C3G as one of the most abundant anthocyanin monomers, offering a cost-effective supplementary source for large-scale C3G extraction and reducing raw material costs for functional product development [23].

## 3. Biosynthesis and Transcriptional Regulation of C3G in *S. nigra*

### 3.1. Biosynthetic Pathway of C3G

**Figure 1:**
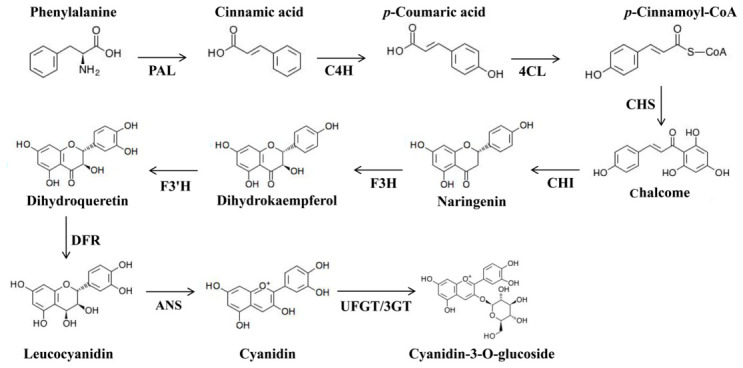
Schematic overview of the cyanidin-3-O-glucoside (C3G) biosynthetic pathway.

The biosynthesis of C3G proceeds via the anthocyanin branch of the flavonoid metabolic pathway, originating from the general phenylpropanoid pathway. Initially, phenylalanine is sequentially converted to 4-coumaroyl-CoA through the catalytic actions of phenylalanine ammonia-lyase (PAL), cinnamate-4-hydroxylase (C4H), and 4-coumarate: CoA ligase (4CL), with PAL serving as the first committed rate-limiting enzyme. Subsequently, one molecule of 4-coumaroyl-CoA condenses with three molecules of malonyl-CoA under the catalysis of chalcone synthase (CHS) to form chalcone, which is stereospecifically cyclized to naringenin by chalcone isomerase (CHI). Naringenin is then hydroxylated by flavanone 3-hydroxylase (F3H) and flavonoid 3′-hydroxylase (F3′H) to produce dihydroquercetin. Dihydroquercetin is subsequently reduced to leucoanthocyanidin by dihydroflavonol 4-reductase (DFR), which is then oxidatively dehydrogenated by anthocyanidin synthase (ANS) to generate the cyanidin cation. Finally, UDP-glucose:flavonoid 3-O-glucosyltransferase (UFGT) transfers a glucose moiety to the 3-hydroxyl group of cyanidin, thereby completing C3G synthesis (Figure 1) [24,25].

### 3.2. Core Regulation of C3G Biosynthesis by the MBW Transcriptional Complex

The C3G biosynthetic pathway is subject to exquisite transcriptional regulation, with the MYB-bHLH-WD40 (MBW) transcriptional complex representing the most extensively characterized regulatory unit. This tripartite complex comprises three classes of transcription factors working synergistically: R2R3-MYB, basic helix–loop–helix (bHLH), and WD40 repeat proteins. In the model plant *Arabidopsis thaliana*, the R2R3-MYB transcription factor PAP1 assembles with GL3/EGL3/TT8 (bHLH) and TTG1 (WD40) proteins to form a functional MBW complex. PAP1 directly activates anthocyanin biosynthetic genes including *DFR*, *ANS*, and *3GT*, and indirectly upregulates *UF3GT*, thereby driving anthocyanin accumulation with C3G as a core component [26]. In tree species research, the TT2-type R2R3-MYB transcription factor JrMYB1L in walnut pericarp forms a classic MBW complex with JrbHLH42 and JrWD40, directly binding to the *JrUFGT* promoter to drive tissue-specific C3G accumulation [27]. In strawberry, FaTRAB1 interacts with FaMYB10 and FaTTG1 to form a novel complex that competitively interferes with canonical MBW complexes, subsequently activating FaF3′H, FaANS, and FaUFGT transcription, redirecting flavonoid metabolic flux, and promoting C3G accumulation through enhanced glycosylation [28]. Notably, the core MBW components governing anthocyanin biosynthesis in *S. nigra* have yet to be cloned and functionally validated; current insights remain extrapolated from model plants or closely related species.

### 3.3. Synthetic Biology and Metabolic Engineering Strategies for Enhancing C3G Production

The detailed elucidation of the C3G biosynthetic pathway and its regulatory mechanisms provides a robust theoretical basis for achieving efficient C3G production through metabolic engineering. For instance, Zong et al. successfully engineered Escherichia coli for the heterologous biosynthesis of cyanidin-3-O-galactoside (a C3G analog) from catechin, thereby opening new avenues for microbial synthesis of C3G-related compounds [29]. In plant metabolic engineering, the overexpression of key MBW complex components—particularly R2R3-MYB transcription factors—has significantly enhanced anthocyanin content in various crops, including apple and tomato [30]. Theoretically, this strategy is applicable to *S. nigra*; however, the current absence of a mature genetic transformation system for this species presents technical bottlenecks that must be overcome for practical implementation.

## 4. Molecular Mechanisms of C3G Antitumor Activity

**Figure 2:**
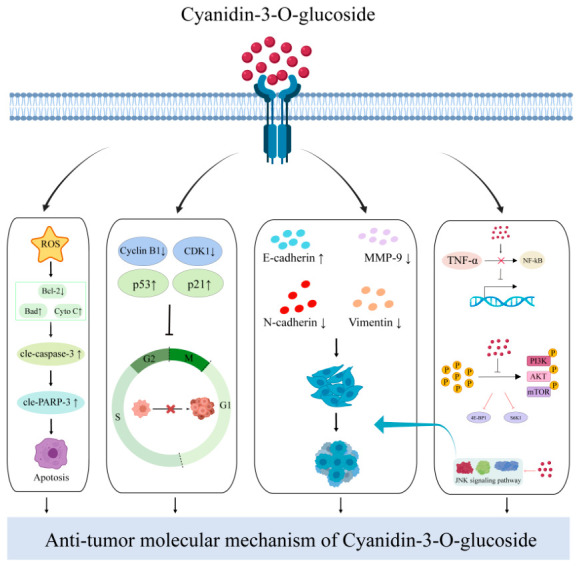
Multifaceted molecular mechanisms underlying C3G-mediated anti-tumor activity.

The antitumor activity of C3G has been evaluated across a spectrum of malignancies, yet the existing evidence remains fragmented and predominantly confined to preclinical models. Table 1 provides a systematic mapping of representative studies by cancer type, evidence level (in vitro or in vivo), and therapeutic modality (monotherapy versus combination therapy). The following subsections then discuss the underlying molecular mechanisms, namely the induction of apoptosis and cell cycle arrest, inhibition of epithelial–mesenchymal transition (EMT) and metastasis, and modulation of key signaling pathways (Figure 2).

### 4.1. Induction of Tumor Cell Apoptosis and Cell Cycle Arrest

In gastric cancer MKN-45 cells, C3G induces apoptosis in a dose- and time-dependent manner through a cascade mechanism that tightly links oxidative stress regulation, mitochondrial dysfunction, and downstream apoptotic signaling pathways [11]. Mechanistically, C3G treatment significantly elevates intracellular ROS levels—a key event in oxidative stress regulation—which in turn triggers the depolarization of the mitochondrial membrane potential, a hallmark of mitochondrial dysfunction. This ROS-mediated mitochondrial impairment leads to an imbalance in Bcl-2 family proteins, characterized by upregulation of the pro-apoptotic protein Bad and downregulation of the anti-apoptotic protein Bcl-2, followed by the release of cytochrome c and the sequential activation of cleaved caspase-3 and cleaved PARP, ultimately executing the apoptotic program [11].

Cell cycle arrest constitutes another critical mechanism by which C3G inhibits tumor cell proliferation. In various tumor cell lines, C3G induces G2/M phase arrest, effectively preventing cells from completing mitosis. Mechanistically, C3G downregulates the expression and activity of cyclin B1 and cyclin-dependent kinase 1 (CDK1) while concurrently upregulating the cell cycle checkpoint proteins p53 and p21, effectively “locking” cells in the G2 phase and blocking their transition to the M phase [9,11,34]. This cell cycle arrest not only directly suppresses rapid tumor cell division but also creates a temporal window for the accumulation of pro-apoptotic signals, thereby establishing synergistic cooperation between apoptosis induction and cell cycle arrest mechanisms.

### 4.2. Inhibition of Epithelial–Mesenchymal Transition and Tumor Metastasis

Epithelial–mesenchymal transition (EMT) is a critical process through which tumor cells acquire migratory and invasive capabilities, representing a core molecular event in tumor metastasis. In an oxaliplatin-resistant colon cancer cell model, C3G has been shown to reverse the EMT process. Through modulation of the Akt/GSK-3β signaling pathway, C3G significantly reduces the migratory capacity of resistant cells and reverses their mesenchymal phenotype. These effects are evidenced by the reversal of E-cadherin downregulation and N-cadherin/vimentin upregulation patterns observed in resistant cells following C3G treatment [31]. These molecular alterations restore the epithelial adhesive phenotype of tumor cells, thereby reducing their capacity to detach from the primary tumor mass and invade surrounding tissues.

C3G also attenuates tumor invasion through the inhibition of matrix metalloproteinase (MMP) activity. MMPs are the primary enzymes responsible for extracellular matrix (ECM) degradation, effectively “clearing pathways” for local tumor infiltration and distant metastasis. Studies demonstrate that C3G treatment significantly reduces MMP-9 expression in tumor cells, thereby suppressing the invasive capacity of multiple tumor cell types [35]. Furthermore, in animal models, C3G effectively suppresses tumor growth and metastatic invasion in vivo by inhibiting JNK signaling pathway-mediated tumor cell autophagy [32].

### 4.3. Modulation of Key Signaling Pathways

#### 4.3.1. NF-κB Signaling Pathway

Nuclear factor-kappa B (NF-κB) is a critical transcription factor regulating inflammation, immune responses, and cell survival. Constitutive NF-κB activation is frequently observed in numerous tumor types, serving as a molecular bridge linking chronic inflammation to carcinogenesis. In intestinal epithelial cells, C3G significantly inhibits TNF-α-induced NF-κB activation and attenuates the expression of downstream target genes, including cyclooxygenase-2 (COX-2) and inducible nitric oxide synthase (iNOS) [16]. COX-2, a key enzyme in prostaglandin synthesis, is closely associated with the development and progression of colorectal cancer and other malignancies, rendering it an important target for C3G-mediated chemoprevention.

#### 4.3.2. PI3K/AKT/mTOR Pathway

The phosphoinositide 3-kinase (PI3K)/AKT/mammalian target of rapamycin (mTOR) signaling pathway represents one of the most critical pro-survival and pro-proliferation pathways in eukaryotic cells, with genes encoding key pathway components frequently mutated or amplified across various human cancers. Chen et al. demonstrated in cervical cancer cells that C3G exerts synergistic effects when combined with the standard chemotherapeutic agent cisplatin, inhibiting cell proliferation while significantly downregulating phosphorylation levels of PI3K, AKT, and mTOR and suppressing the activation of downstream target proteins S6K and 4E-BP1 [10]. The mechanisms underlying this synergy are twofold: first, C3G-mediated inhibition of the PI3K/AKT/mTOR pathway weakens tumor cell anti-apoptotic defenses, thereby sensitizing cells to cisplatin-induced cell death; second, the antioxidant properties of C3G mitigate cisplatin-induced oxidative damage in normal tissues, effectively broadening the therapeutic window.

#### 4.3.3. JNK and MAPK Pathways

c-Jun N-terminal kinase (JNK) and mitogen-activated protein kinase (MAPK) family members play pivotal roles in cellular stress responses, apoptosis regulation, and tumor development. Chen et al. confirmed in animal tumor models that C3G suppresses tumor growth and metastatic invasion in vivo by inhibiting JNK signaling pathway-mediated tumor cell autophagy [32]. Additionally, in an SKH-1 hairless mouse skin model, C3G effectively inhibited UVB-induced oxidative damage and inflammatory responses through the modulation of MAPK and NF-κB signaling pathways, suggesting its potential utility in skin cancer chemoprevention [33].

## 5. Association Between C3G In Vivo Metabolism and Antitumor Activity

### 5.1. Absorption and Distribution in the Human Body

The pharmacokinetic profile of C3G in humans directly determines whether effective concentrations can be achieved in target tissues to elicit antitumor effects. Current evidence highlights the importance of integrating gut microbiota- and hepatic-mediated biotransformation into the pharmacological evaluation of natural products, particularly those with low oral bioavailability [36]. As early as 1999, Cao et al. detected anthocyanins in the plasma of subjects following oral administration of elderberry extract, providing the first direct evidence that C3G from *S. nigra* is absorbable in humans [37]. Subsequent pharmacokinetic and mechanistic studies have elucidated that C3G is absorbed in its unmodified form in the upper digestive tract and its metabolites rapidly enter the systemic circulation [7]. Notably, C3G is absorbed in its intact glycosylated form—a finding that challenged the previous prevailing assumption that anthocyanins must undergo intestinal deglycosylation prior to absorption [7]. However, the absolute oral bioavailability of C3G remains extremely low, typically reported to be below 1%, indicating that the vast majority of ingested C3G fails to enter systemic circulation [17].

### 5.2. Gut Microbiota-Mediated Biotransformation and Antitumor Activity of Metabolites

Although a fraction of C3G is absorbed in the small intestine, the majority of the ingested dose reaches the colon, where it undergoes extensive metabolic transformation mediated by the gut microbiota. Intestinal bacteria employ enzymatic reactions, prominently β-glucosidase hydrolysis, to convert C3G into various low-molecular-weight phenolic metabolites, primarily including protocatechuic acid, ferulic acid, vanillic acid, and phloroglucinaldehyde [38].

Protocatechuic acid, the predominant microbial metabolite of C3G, exerts anti-tumor effects through mechanisms that closely parallel those of its parent compound. This metabolite induces apoptosis across multiple cancer types, including breast, lung, liver, cervical, and prostate cancer cells [39]. Mechanistically, protocatechuic acid disrupts cellular redox homeostasis by downregulating heme oxygenase-1 and upregulating p21, leading to oxidative stress and apoptosis in colon cancer cells [40]. Furthermore, it suppresses lung cancer cell migration and invasion by blocking the PI3K/Akt/mTOR signaling pathway and reversing epithelial–mesenchymal transition [41]. These signaling pathways—PI3K/Akt/mTOR and oxidative stress-mediated apoptosis—are the same as those targeted by C3G [10,11], supporting a “parent–metabolite synergy” model wherein the in vivo antitumor efficacy of C3G results from the combined actions of the parent compound and its key metabolite, protocatechuic acid.

Collectively, the antitumor effects of C3G in vivo likely result not solely from the parent compound but from the synergistic action of C3G and its microbial metabolites, particularly protocatechuic acid. This “parent compound–metabolite synergy” model is critical for understanding C3G biological effects in vivo: even when C3G plasma concentrations are low, its metabolites can maintain effective concentrations locally in the intestinal lumen and in the systemic circulation, collectively contributing to antitumor activity.

### 5.3. Challenges and Strategies for Improving C3G Bioavailability

The low oral bioavailability of C3G represents a major bottleneck limiting its translation from functional food ingredients to clinical therapeutics. Key factors contributing to this limitation include poor permeability across intestinal epithelial cells, rapid hepatic first-pass metabolism, and efficient elimination via biliary secretion and urinary excretion. Furthermore, C3G is highly susceptible to ring-opening and oxidative degradation in the neutral-to-weakly alkaline environment of the small intestine and colon, further restricting its effective absorption [17].

To overcome these bioavailability limitations, researchers have developed various advanced delivery strategies. Devi et al. employed albumin-decorated nanostructured lipid carriers (NLCs) to deliver polyphenol-rich *S. nigra* extract, leveraging the enhanced permeability and retention (EPR) effect to improve tumor-targeted drug accumulation [42]. In parallel, the same group prepared sulfated C3G and its metabolites through chemical hemisynthesis and identified endogenous sulfated products through analysis of human urine samples, providing important tool compounds for investigating C3G metabolism in vivo [43]. Nevertheless, current delivery strategies remain under active optimization; achieving enhanced bioavailability while simultaneously ensuring carrier biocompatibility, stability, and manufacturing scalability represents a core challenge that must be resolved to advance C3G toward clinical applications.

## 6. Challenges and Future Perspectives

### 6.1. Core Challenges in Current Research

Despite significant advances in *S. nigra* C3G research, several critical challenges must be addressed to facilitate the successful transition from basic research to clinical application.

A primary bottleneck limiting the directed enhancement of C3G content in *S. nigra* is the absence of species-specific functional genomics data. Unlike model plants where MBW complex members have been cloned and their target gene binding specificity resolved, *S. nigra* lacks validated transcriptional regulators, making it impossible to predict the efficacy of cisgenic or transgenic strategies. This molecular blind spot, combined with the absence of a stable genetic transformation protocol for this species, renders metabolic engineering efforts largely speculative.

High-quality clinical evidence is severely lacking. The vast majority of C3G antitumor studies remain confined to in vitro cell-based experiments and preclinical animal models; randomized controlled clinical trials involving cancer patients or high-risk populations are virtually nonexistent. Whether C3G exerts antitumor effects in vivo through the molecular mechanisms already elucidated (e.g., NF-κB, PI3K/AKT/mTOR pathway inhibition) requires rigorous validation through in vivo imaging, target occupancy studies, and genetically engineered animal models.

### 6.2. Future Perspectives

#### 6.2.1. In-Depth Elucidation of C3G Biosynthetic Regulatory Networks in *S. nigra*

Future efforts should move beyond cross-species inference by establishing an *S. nigra*-specific functional genomics platform. Priority should be given to (i) transcriptome-guided cloning of R2R3-MYB, bHLH, and WD40 orthologs from high-C3G genotypes; (ii) yeast two-hybrid and BiFC assays to reconstruct native MBW interaction networks; and (iii) transient or stable transformation (e.g., Agrobacterium-mediated hairy root or protoplast systems) to validate target gene binding specificity and transcriptional activation capacity. Integrating these data with metabolomic quantification will enable predictive modeling of metabolic flux redirection, ultimately providing molecular targets for CRISPR-based promoter editing or elite germplasm marker-assisted selection.

#### 6.2.2. Advancing In Vivo Validation and Clinical Translation of C3G Antitumor Mechanisms

Current C3G antitumor research remains predominantly based on in vitro cell experiments and conventional animal models, with a critical shortage of high-quality clinical evidence necessary to support clinical translation. Future investigations should employ patient-derived tumor organoids and advanced gene-edited animal models that more faithfully recapitulate the human tumor microenvironment to validate the in vivo regulatory effects of C3G on key signaling pathways, including NF-κB, PI3K/AKT/mTOR, and JNK. Concurrently, well-designed randomized controlled clinical trials targeting high-risk populations and postoperative adjuvant therapy patients are essential to establish safe dosage ranges, optimal treatment cycles, and meaningful efficacy endpoints, and to rigorously evaluate the efficacy and toxicity profiles of C3G as monotherapy or in combination with standard chemotherapeutic agents (e.g., cisplatin, oxaliplatin). Furthermore, advanced in vivo imaging modalities and target occupancy analysis should be employed to directly visualize C3G distribution in tumor tissues and definitively identify its molecular targets, thereby providing critical data support for the precision clinical application of C3G.

## Figures and Tables

**Table 1:** Comparative overview of C3G anti-tumor studies across cancer types.

Cancer Type	Evidence Level	Combination Therapy	Reference
Melanoma	In vitro + In vivo	Monotherapy	[9]
Cervical cancer	In vitro	Combination with cisplatin	[10]
Gastric cancer	In vitro	Monotherapy	[11]
Colorectal cancer	In vitro	Combination with oxaliplatin	[31]
Malignant tumor	In vivo	Combination with chloroquine	[32]
Skin cancer	In vivo	Monotherapy	[33]

## Data Availability

No new data were created or analyzed in this study.

## Abbreviations

The following abbreviations are used in this manuscript:

AKT	Protein kinase B
ANS	Anthocyanidin synthase
Bcl-2	B-cell lymphoma 2
BCRP	Breast cancer resistance protein
C3G	Cyanidin-3-O-glucoside
CDK	Cyclin-dependent kinase
COX-2	Cyclooxygenase-2
ECM	Extracellular matrix
EMT	Epithelial–mesenchymal transition
EPR	Enhanced permeability and retention
FW	Fresh weight
GLUT2	Glucose transporter 2
GSK-3β	Glycogen synthase kinase-3 beta
iNOS	Inducible nitric oxide synthase
JNK	c-Jun N-terminal kinase
MAPK	Mitogen-activated protein kinase
MMP	Matrix metalloproteinase
mTOR	Mammalian target of rapamycin
NF-κB	Nuclear factor-kappa B
NLCs	Nanostructured lipid carriers
Nrf2	Nuclear factor erythroid 2-related factor 2
PCA	Protocatechuic acid
PI3K	Phosphoinositide 3-kinase
ROS	Reactive oxygen species
SGLT1	Sodium-glucose linked transporter 1
TNF-α	Tumor necrosis factor-alpha
UDP	Uridine diphosphate
